# Use of multiple traits genomic prediction, genotype by environment interactions and spatial effect to improve prediction accuracy in yield data

**DOI:** 10.1371/journal.pone.0232665

**Published:** 2020-05-13

**Authors:** Hsin-Yuan Tsai, Fabio Cericola, Vahid Edriss, Jeppe Reitan Andersen, Jihad Orabi, Jens Due Jensen, Ahmed Jahoor, Luc Janss, Just Jensen

**Affiliations:** 1 Center for Quantitative Genetics and Genomics, Aarhus University, Tjele, Denmark; 2 Department of Marine Biotechnology and Resources, National Sun Yat-Sen University, Kaohsiung, Taiwan; 3 Rijk Zwaan, De Lier, Netherlands; 4 Nordic Seed, Galten, Denmark; 5 Department of Plant Breeding, Swedish University of Agricultural Sciences, Alnarp, Sweden; Institute of Genetics and Developmental Biology Chinese Academy of Sciences, CHINA

## Abstract

Genomic selection has been extensively implemented in plant breeding schemes. Genomic selection incorporates dense genome-wide markers to predict the breeding values for important traits based on information from genotype and phenotype records on traits of interest in a reference population. To date, most relevant investigations have been performed using single trait genomic prediction models (STGP). However, records for several traits at once are usually documented for breeding lines in commercial breeding programs. By incorporating benefits from genetic characterizations of correlated phenotypes, multiple trait genomic prediction (MTGP) may be a useful tool for improving prediction accuracy in genetic evaluations. The objective of this study was to test whether the use of MTGP and including proper modeling of spatial effects can improve the prediction accuracy of breeding values in commercial barley and wheat breeding lines. We genotyped 1,317 spring barley and 1,325 winter wheat lines from a commercial breeding program with the Illumina 9K barley and 15K wheat SNP-chip (respectively) and phenotyped them across multiple years and locations. Results showed that the MTGP approach increased correlations between future performance and estimated breeding value of yields by 7% in barley and by 57% in wheat relative to using the STGP approach for each trait individually. Analyses combining genomic data, pedigree information, and proper modeling of spatial effects further increased the prediction accuracy by 4% in barley and 3% in wheat relative to the model using genomic relationships only. The prediction accuracy for yield in wheat and barley yield trait breeding, were improved by combining MTGP and spatial effects in the model.

## Introduction

Wheat (*Triticum aestivum* L.) and barley (*Hordeum vulgare* L.) are two of the earliest domesticated crop species and are ranked as the first and fourth most-grown cereals worldwide, respectively [1–4]. Approximately 75% of barley’s global production is used as an ingredient in animal feed with the remaining 25% used for alcoholic and non-alcoholic beverages and a variety of other foodstuffs. Due to barley’s diploid genome architecture and its ability to self-fertilize, barley is considered an ideal model species for cereal genetic research [5]. Most wheat varieties are tetraploid (durum) or hexaploid (bread), but a few diploid varieties also exist. Due to their importance in food production, a high quality assembly of the entire genome sequence for barley is publicly available [1]. In contrast, the first genome assembly for wheat became available only recently [4], enhancing the opportunities for plant breeders to advance genome-assisted crop improvements and discover quantitative trait loci (QTLs) of commercial interest.

Previous researchers have indicated that most traits of commercial importance in barley and wheat (*e*.*g*., yield) can likely be explained by many QTLs, each of which provide small contributions to total genetic variance [6,7]. This architecture has significantly restricted the application of traditional marker-assisted selection techniques, particularly for economically important traits with a highly polygenic architecture. The concept of genomic selection (GS) proposed by Meuwissen et al. [8] was developed to incorporate whole-genome marker data in selection programs to accumulate single nucleotide polymorphisms (SNP) or haplotype effects that can accurately predict future performance of potential new lines. As such, genomic prediction (GP) is now utilized to predict the breeding values of individuals based on a sufficient number of molecular markers and a training population (TP) that is genotyped and phenotyped for traits of interest. The performances of phenotypes in a validated population (VP) can then be predicted by exploiting dense molecular markers (or QTLs) that are associated with traits in the TP. For commercial breeding programs, large scale phenotyping and genotyping of breeding lines in the TP can lead to the development of promising statistical models for variance component estimation and for predicting breeding values using established approaches (e.g., REML [9] and BLUP [10]). In contrast to animal breeding approaches, the utilization of genomic approaches in plant breeding has been developed only recently [11].

Several methods in statistical genetics have been developed that benefit from genetic correlations between traits [12–14]. Univariate analysis, also known as single trait genomic prediction (STGP), is currently the most common method used in plant breeding schemes (e.g., in cassava [15], wheat [16–18], barley [19], rye [20], and rice [21]). However, for most commercial plant breeding programs, breeders have collected data on several phenotypes, which enable them to take advantage of genetic and phenotypic correlations among traits. Such multiple trait genomic prediction (MTGP) methods have recently been extensively examined [15,17,18,22,23].

The MTGP approach was originally developed to exploit information gained from correlated indicator traits [13]. Results have generally indicated that MTGP can increase the accuracy of genetic evaluations, especially when traits with high genetic correlations are involved in the analyses [13,15,17,20,22–24]. These findings agree with expected advantages of indirect selection [25]. Compared with traditional pedigree-based breeding methods and STGP, MTGP will likely be able to provide an ideal alternative for characterizing a higher number of candidate genes for selection and at lower cost, especially for traits that are labor intensive to evaluate or require a long time before they are expressed (e.g., baking quality or resistance to pests).

For several economic traits of spring wheat, studies have shown that correlations between observed phenotypes and estimated breeding values are higher when the genomic prediction model involves both genomic and pedigree information than when pedigree alone is used [26]. In general, commercial plant breeders usually have phenotypic records across multiple generations for traits of economic importance. In this study, we used data from multiple plots of F_5_ generations and analyzed those results jointly with records from replicated experiments of F_6_ generations from a variety of field locations. Because testing conditions are not necessarily identical for each generation, it may be necessary to treat records from different generations as being different, but correlated traits. This approach might considerably increase selection accuracy and further increase the genetic gain achieved per generation [15,17].

The major aims of this study were to: (1) compare the predictive ability for both genomic information and spatial effect in breeding lines of winter wheat and spring barley, (2) evaluate the prediction accuracy underlying STGP and MTGP methods, and (3) apply F_5_ and F_6_ data in the MTGP analysis (as multiple training populations) to predict the future yield in breeding lines of winter wheat and spring barley.

## Materials and methods

### Field experiment and phenotypes

**Fig 1:**
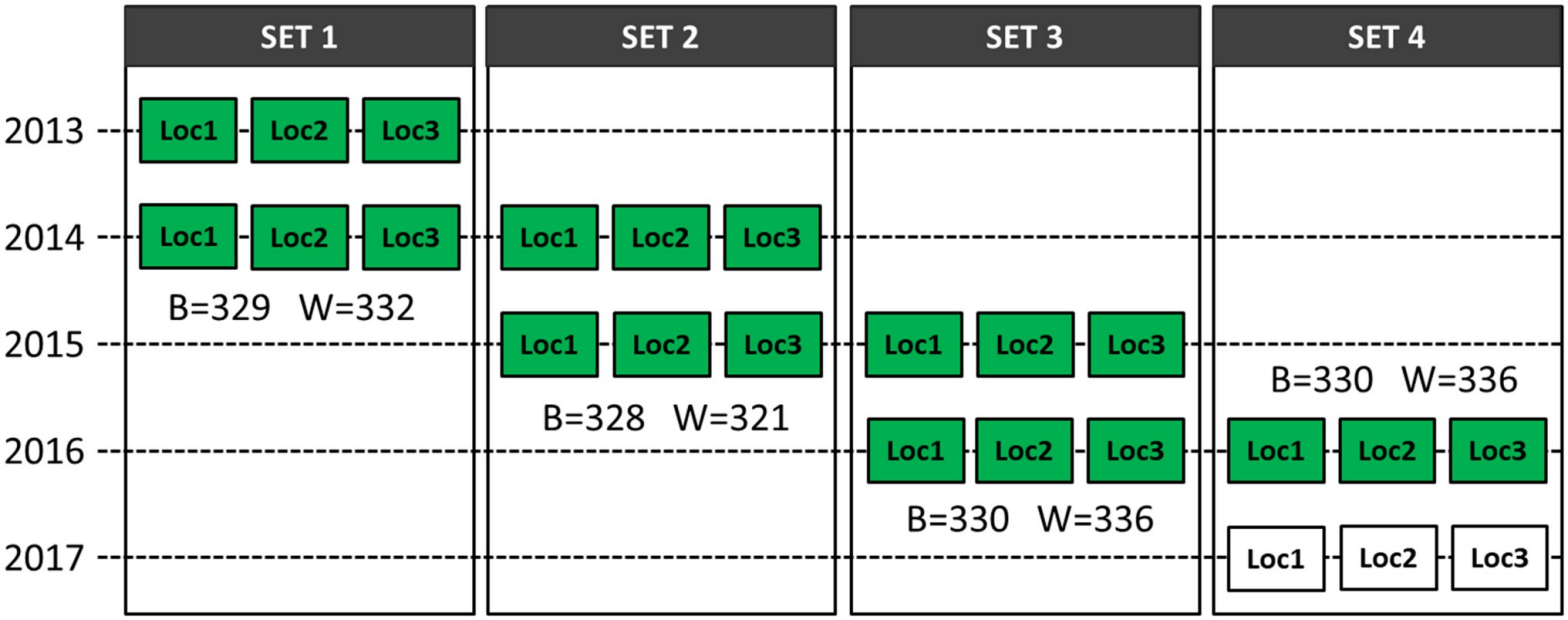
Trial plan of spring barley and winter wheat field growth experiments. ‘B’ is the number of lines in spring barley in each corresponding set and ‘W’ is the number of lines in winter wheat. Each set contains data from two consecutive years. For instance, set 1 contained data from 2013 to 2014, set 2 contained data from 2014 to 2015, and so on. The green box represents data we included in the test, whereas the white box in Set 4 represents data still under collection at time of analysis, and not yet included in the test. The figure was adapted from and originally drawn by Andrea Bellucci (pers. comm.).

Our field experiment was performed by Nordic Seed A/S (Galten, Denmark). In total, we tested 1,317 spring barley (*H*. *vulgare*) and 1,325 winter wheat (*T*. *aestivum*) breeding lines. We tested each line in two consecutive years and at three locations every year (Fig 1). The three locations tested were Dyngby, Holeby, and Skive (for first year only) in Denmark.

We nested multiple trials within the three test locations, and tested plots using a randomized complete block design [27] within each trial and each trial contained the same number of breeding lines. For barley, every trial comprised 22 lines and 3 checks, with 3 replicates in the first year and 2 replicates in the second year. For wheat, every trial had 21 lines and 4 checks, with 2 replicates in each year.

For each trial, lines from any given family were sown in a randomized order, in each replicate, next to each other in the field. Based on the size of the family, a trial consisted of one or more families, and if the last family to be sown was more numerous than the remaining available plots, they were sown in the next trial. Therefore, many families had members in at least two different trials. In general, there were 3–5 full-siblings in each trial.

Yield data for the F_5_ and F_6_ generations were collected in this study. Every year, we made a new set of crosses and every set contained approximately 330 unique single seed descent lines in F_5_, which were then used to produce the F_6_ line. The number of recorded plots in F_6_ were slightly different in spring barley and winter wheat, as details in Table 1. The yield data were measured as kg grain per 8.25-m^2^ plot in both spring barley and winter wheat breeding lines for F_5_ and F_6_, respectively.

The phenotypes of trait and pedigree information of every line were recorded by Nordic Seed A/S (Galten, Denmark). The three farms used are owned by Nordic Seed A/S, and they, therefore did not need any further permission to use the land. The three farms are legal for farming use, and not located on any national parks or other protected areas of land or sea.

**Table 1:** Descriptive statistics for spring barley and winter wheat phenotypic records.

Species	Trait	Units	No. of Plots	Mean (SD)	Min.	Max.
Barley	Yield F_6_	kg grain /8.25m^2^ per plot	15376	6.60 (0.8)	4.2	9.4
	Yield F_5_		1317	6.11 (1.0)	3.7	8.0
Wheat	Yield F_6_		13329	8.62 (0.9)	3.9	14.8
	Yield F_5_		1325	9.68 (1.8)	4.1	13.4

### Genotypes

We used the Illumina 9K barley SNP-chip and the 15K wheat SNP-chip to genotype all breeding lines. After quality control procedures, 4,056 SNPs in spring barley and 11,154 SNPs in winter wheat remained for analysis using the following two filters: (1) a minor allele frequency of <0.01 and (2) a missing SNP frequency per line value of >0.02. There were 2,841 SNPs in spring barley and 9,290 SNPs in winter wheat mapped to existing linkage groups according to the genome assembly [1,4], whereas 1,215 SNPs in spring barley and 1,864 SNPs in winter wheat had unknown positions in the genome.

### Statistical methods

Pedigree relationship matrices were constructed based on the pedigree information of spring barley and winter wheat using the tabular approach [28,29], which assumed that parental lines have nine cycles of self-fertilization. Genomic relationship matrices (**G**) were generated for spring barley and winter wheat, using the first method of VanRaden (2008) [30], with **G = ZZ’ /** 2**∑***p*_***j***_ **(**1**-***p*_***j***_**)**, where the matrix **Z** was calculated as (**M**–**P**). **M** is a matrix of minor allele counts (0, 1, and 2) with *m* columns (one for each marker) and *n* rows (one for each line). **P** is a matrix containing allele frequencies, with column *j* defined as **l2(***p*_*j*_ − 0.5**)**, wherein **l** is a vector of ones, and *p*_*j*_ is the frequency of the second allele at corresponding locus *j*. After quality control procedures, the percentage of missing values was about 1% for both species in the genotype file before the genomic relationship matrices were constructed. The mean imputation approach was then applied to assign any missing genotypes [30]. All the missing genotypes were imputed while constructing the genomic relationship matrices. We performed a principle coordinate analysis (PCoA) (Fig 3) on the genomic relationship matrix using the built-in R function [31]. We used univariate and multivariate linear mixed models to obtain REML estimates of the variance components of traits using the DMU multivariate mixed model package [32].

### Model used for yield traits of F_5_ and F_6_ generations

We developed the following models for the analyses. Model 1 was developed for yield for both F_5_ and F_6_ generations using only genomic information (**G**). As yield data were both available for F_5_ and F_6_ in spring barley and winter wheat, the univariate and multivariate analyses were applied using Model 1:

$$y=Xb+Z_{1}g+e$$ (1)

The **b** is the fixed factor comprising year, location, and trial (YLT), whereas the **g** is the genomic information. In addition, to estimate effects from pedigree information and spatial effects, we also developed Model 2 for F_5_ and F_6_ yields. The **b** and **g** terms are described by Model 2:

$$y=Xb+Z_{1}a+Z_{2}g+\sum_{i=1}^{n}Z_{i}s+e$$ (2)

where the **a** term corresponds to additive genetic effects using pedigree information for the covariance structure, the **s** term is a spatial effect variable to account for local spatial variation of experiments in the field.

**Fig 2:**
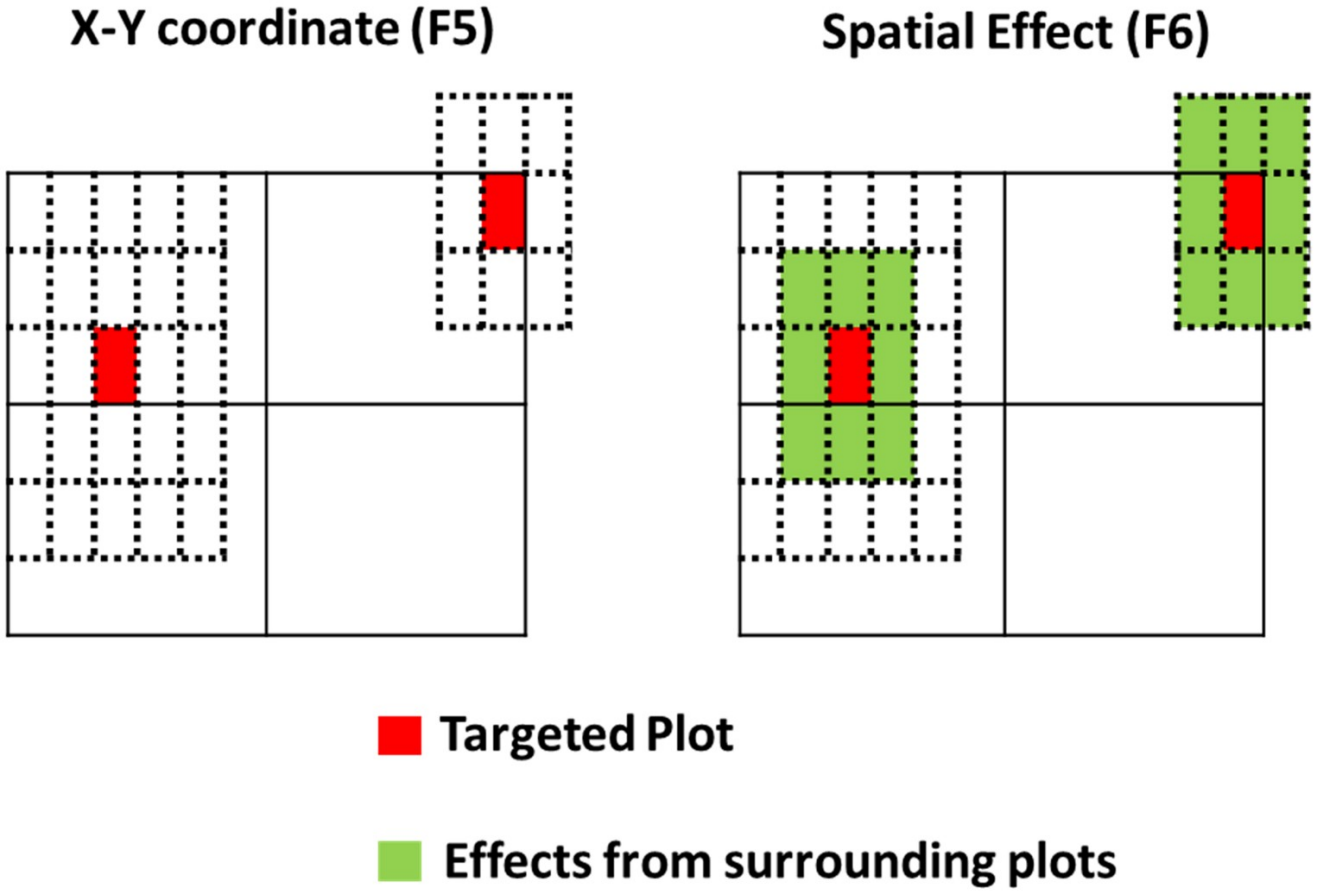
Illustration of spatial effects employed in the F_5_ and F_6_ test. In the F_5_ test, we fitted X- and Y-coordinates as the spatial effect in the model, whereas for the F_6_ test, we included its eight surrounding plots as well in the spatial effect (as a moving average). The figure was adapted from and originally drawn by Andrea Bellucci (pers. comm.).

For the models described above, where **y** is a vector of observations for one trait, **X** is a design matrix for the fixed effect, and the **b** term is the vector of fixed effects, including combined effects of year, location, and trial (YLT). **Z**_**n**_ comprises the design matrices of random effects and the **g** term is a vector of additive genetic effects with $g\sim \text{N}(0,G\sigma_{g}^{2})$, wherein $\sigma_{g}^{2}$ represents genomic variance and **G** is the genomic relationship matrix. The distribution of $a\sim \text{N}(0,A\sigma_{a}^{2})$, then $\sigma_{a}^{2}$ represents the additive genetic variance and **A** is the pedigree relationship matrix. The **s** term is a vector of spatial effect with $s\sim \text{N}(0,I\sigma_{s}^{2})$, which contains the X and Y coordinates of plots in the F_5_ test (n = 2), and eight surrounding plots and plot itself in the F_6_ test (n = 9), as illustrated in Fig 2. The **e** term is a vector of random residuals with $e\sim \text{N}(0,I\sigma_{e}^{2})$.

For multivariate analysis, we modeled two traits together to estimate all effects, including the marker effects. The testing combinations are detailed in Fig 4. Taking Model 1 as an example, **y**_**1**_ represents yield for F_6_ and **y**_**2**_ represents yield for F_5_. The year, location, and trial (YLT) serves as a fixed factor represented by **b**_**n**_ in the model. The terms **X**_**n**_ and **Z**_**n**_ are the designed matrices of the fixed factor and random factor, respectively. The **g**_**n**_ term is the genomic information, as described in the statistical model section. We assumed the residual covariance to be zero because yield in F_5_ and F_6_ generation were statistically independent (as they were collected from different years and generations).

$$\left[\begin{array}{c} y_{1}\\ y_{2} \end{array}\right]=\left[\begin{array}{cc} X_{1} & 0\\ 0 & X_{2} \end{array}\right]\left[\begin{array}{c} b_{1}\\ b_{2} \end{array}\right]+\left[\begin{array}{cc} Z_{1} & 0\\ 0 & Z_{2} \end{array}\right]\left[\begin{array}{c} g_{1}\\ g_{2} \end{array}\right]+\left[\begin{array}{c} e_{1}\\ e_{2} \end{array}\right]$$ (3)

where $\left[\begin{array}{c} g_{1}\\ g_{2} \end{array}\right]\sim \text{N}(0,G⊗H)$ with $H=\left[\begin{array}{cc} \sigma_{g1}^{2} & \sigma_{g12}\\ \sigma_{g12} & \sigma_{g2}^{2} \end{array}\right]$, wherein **H** is the variance and covariance matrix of the genomic breeding values of the two traits, and for $\left[\begin{array}{c} e_{1}\\ e_{2} \end{array}\right]\sim \text{N}(0,I⊗R)$ with $R=\left[\begin{array}{cc} \sigma_{e1}^{2} & \sigma_{e12}\\ \sigma_{e12} & \sigma_{e2}^{2} \end{array}\right]$, **R** is the residual variance and covariance matrix of the two traits. Residual co-variance did not exist when we performed yield calculations for F_5_ and F_6_ generations, because the traits were collected from different years. When there were missing data for one of the traits, the residual variance was equal to $\sigma_{e}^{2}$ for the observed trait.

We used the variances to estimate the heritability of line means. The total phenotypic variance ($\sigma_{p}^{2}$) of line means was:

$$\sigma_{p}^{2}=d\left(G\right)\sigma_{g}^{2}+\frac{n_{s}\sigma_{s}^{2}}{r_{1}}+\frac{\sigma_{e}^{2}}{r_{2}}$$ (4)

Heritability was estimated as:

$${\text{h}}^{2}=\text{d}(G)\sigma_{g}^{2}/\sigma_{p}^{2}$$ (5)

where d(**G**) is the mean diagonal element of the genomic relationship matrix, *n*_*s*_ is the number of surrounding plots considered in the spatial effect, *r*_*n*_ is the number of replicates of corresponding effects for each genotype when estimating line heritability, and *r*_*n*_ was one (1.0) when estimating the narrow-sense plot heritability [33] based on the data of a single plot. The narrow-sense plot heritability was used to consider the random effects from the plot itself, whereas the line heritability was used to calculate the mean of effects from records across all replicates based on the same breeding line [6]. Line heritability is higher than plot heritability when there are more replicates in the experiment.

### Cross-validation and predictive ability

For our multivariate analysis (MTGP) of the F_5_ and F_6_ yield dataset in particular, we used four sets (Sets 1, 2, 3, and 4) in F_5_ as the first trait, and Sets 1, 2, and 3 in F_6_ as the second trait to predict the yield performance of Set 4 in F_6_. This strategy helped us test the feasibility of using a multivariate analysis for predicting future traits of interest in the coming year. For univariate analysis (STGP), we used Sets 1, 2, and 3 for yield by F_6_ data as the training population to predict Set 4 for yield by F_6_ (as a validation population). We estimated the predictive ability for future yield performance [ρ(**ӯ**_**c**_, **ĝ**)] by calculating the correlation between the average of phenotypic records corrected for the fixed effect (**ӯ**_**c**_) and genomic predicted breeding values (**ĝ**). The accuracy of predicting additive breeding values we calculated as the predictive ability divided by the square root of heritability of line means: ρ(**ӯ**_**c**_, **ĝ**)/h.

## Results

### Genomic relationship analysis among breeding lines

**Fig 3:**
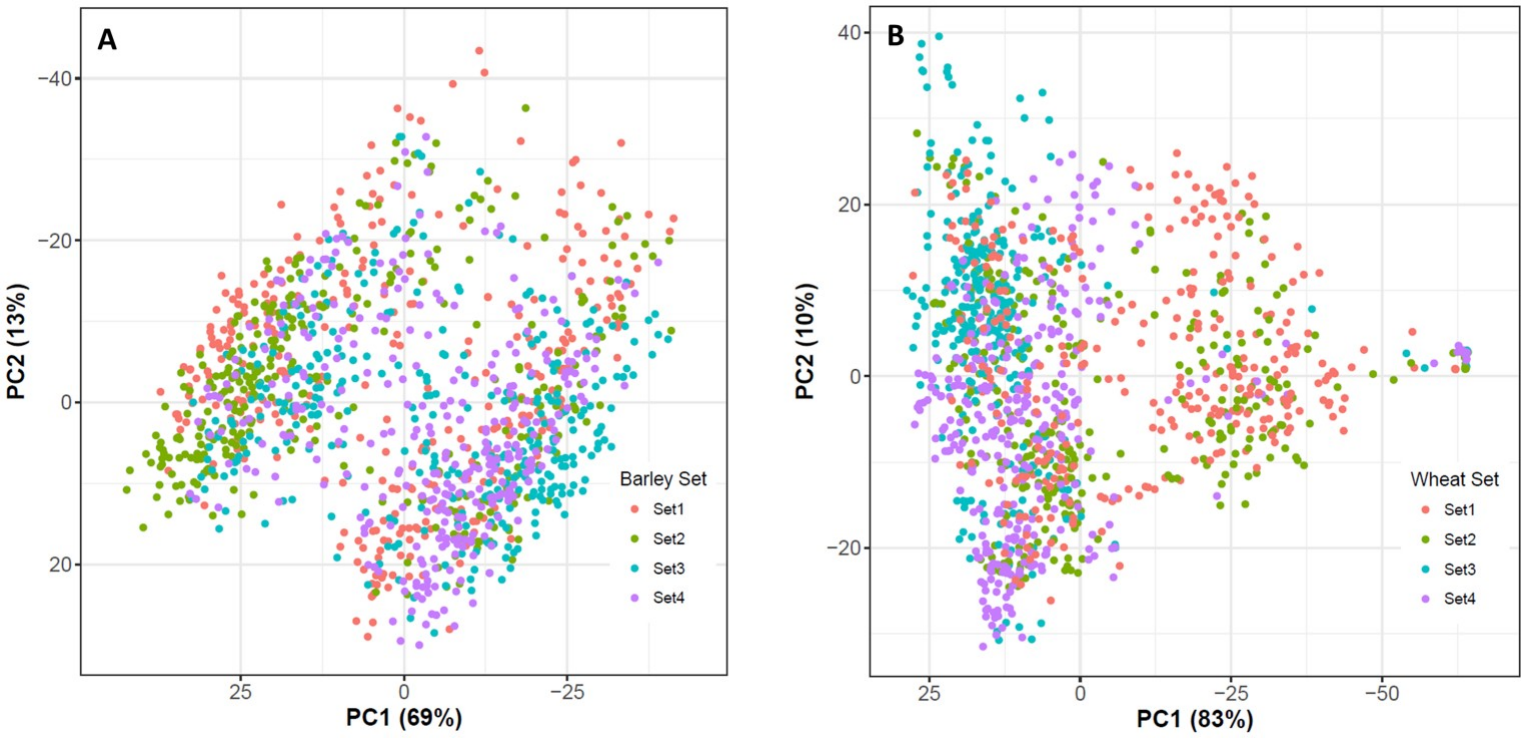
Principal coordinate analysis of (a) spring barley and (b) winter wheat.

The first two principal components of PCoA explained 69% (Axis 1) and 13% (Axis 2) of the total variance in genomic relationships for spring barley, and 83% (Axis 1) and 10% (Axis 2) for winter wheat (Fig 3). In general, most lines were highly genetically associated with others. Based on genomic information, PCoA indicated that there were clearly identifiable groups in spring barley and winter wheat, implying that certain lines were from the same groups. For example, there were some lines from Set 2 segregating in the left area of the PCoA plot in barley, whereas Set 3 also segregated in left area of the PCoA plot in wheat. However, in general, although the PCoA plot showed that there were only two major genetic clusters in both species, we also found that certain lines came from different crosses, sets, and parents. The heat-map of genomic relationship (using a similar dataset) also highlights the same results for both grain species [6,7].

### Descriptive statistics and variance components

We studied yield traits in spring barley and winter wheat commercial breeding lines. The number of plots and phenotype statistics for each trait are listed in Table 1. The heritability and variance component estimates of traits are given in Table 2. Heritability (using the genomic-based method) of yield in F_5_ was 9% for spring barley and 41% for winter wheat, whereas the heritability of yield in F6 was 24% for spring barley, and 33% for winter wheat (Table 2).

**Table 2:** Variance components, narrow-sense plot heritability, and correlation estimation of traits using model 1. The column for $\sigma_{g}^{2}$ and $\sigma_{e}^{2}$ are given by 10^−2^ as base unit.

Species	Traits	$\sigma_{g}^{2}$(x 10^−2^)	$\sigma_{e}^{2}$(x 10^−2^)	plot^1^ h^2^	line h^2^	Cor_G^3^
Barley	Yield F_5_	0.3	6.6	0.09	0.09	0.7
	Yield F_6_	1.7	5.7	0.24	0.75	
Wheat	Yield F_5_	2.9	7.8	0.41	0.41	0.72
	Yield F_6_	7.6	22.8	0.33	0.76	

^1^ The plot heritability. For yield F_5_, we only have one plot in F_5_, so r_n_ in the denominator is always one (see Model 4) and the plot heritability is equal to line heritability. For other traits, we have multiple plots from the same breeding line, so we obtained more information based on the same breeding line. Therefore, line heritability is higher than plot heritability. See more descriptions in Model 4.

^2^ Line heritability.

^3^ The environmental correlation was set as independent between yield F_6_ and F_5_ because their records were collected in different years and locations. Therefore, only genetic correlations (Cor_G) are provided for yield traits.

### STGP versus MTGP

**Fig 4:**
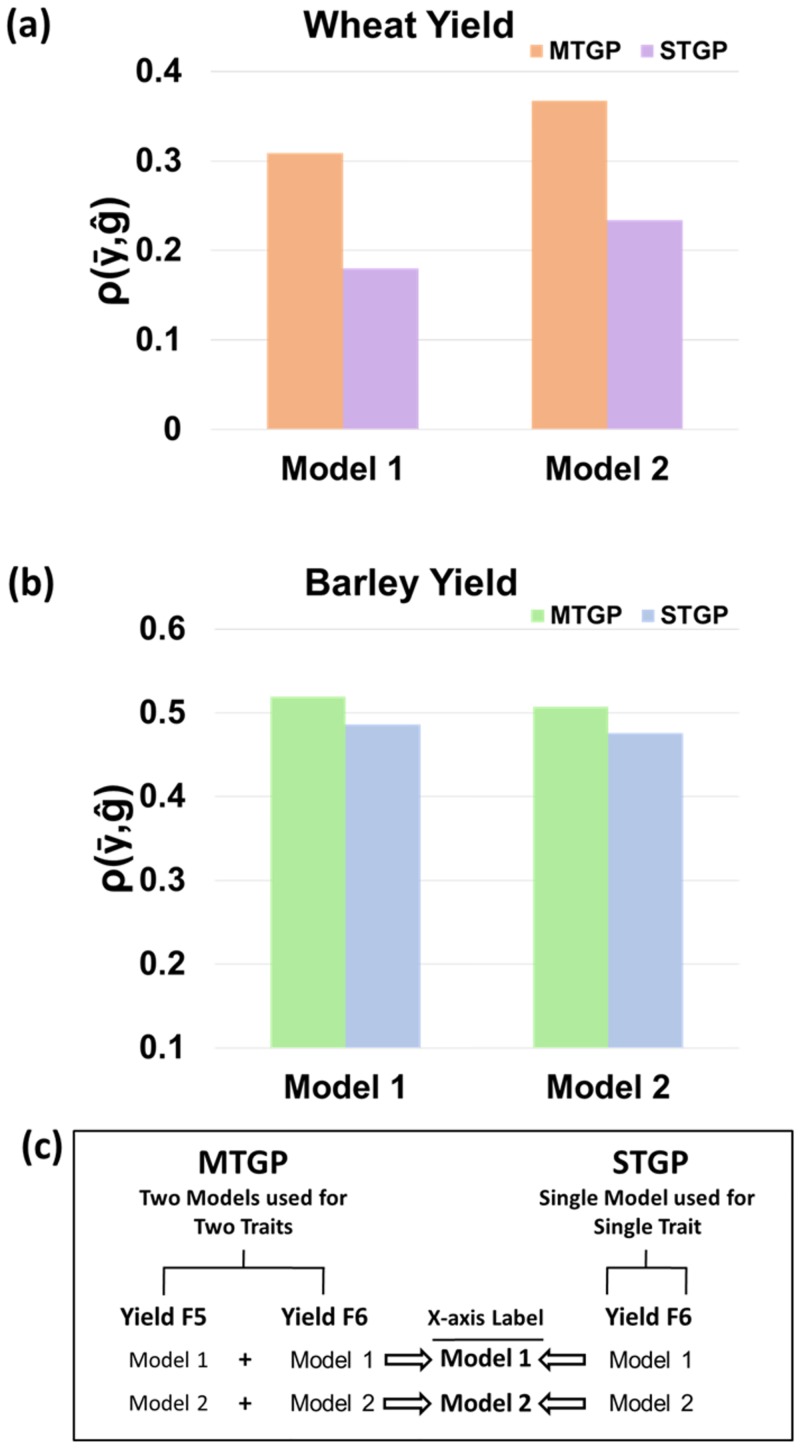
Comparison of MTGP and STGP approaches for predicting yield in the F_6_ generation of winter wheat and spring barley. For MTGP, we used a training population, including F_5_ as Trait I, Sets 1, 2, 3 for yield by F_6_ as Trait II, and Set 4 to predict yield of F_6_ (as a validation population). For STGP, we used Sets 1, 2, and 3 for yield by F_6_ data as the training population to predict Set 4 for yield by F_6_ (as a validation population). Fig 4c shows the corresponding models used for MTGP and STGP, respectively. The corresponding models are described in statistical model section in material and methods.

For multiple trait analysis, we used yield in F_5_ as the first trait and yield in F_6_ as the second trait to predict the future yield performance for the spring barley and winter wheat breeding lines. Overall, the prediction accuracies of bivariate analyses were higher than for univariate analyses in all scenarios (Fig 4). In using a bivariate analysis, we improved predictive accuracies by about 7% in spring barley and by about 57% in winter wheat varying from STGP and MTGP. The MTGP model that combined pedigree, the genomic relationship matrix, and spatial effects showed higher prediction accuracy than using the genomic relationship matrix only.

## Discussion

The goal of this study was to utilize pedigree information, genomic information, and genetic covariance between associated traits to increase the accuracy of prediction of economically important traits in cereal breeding programs. Our main findings were that the prediction accuracy of yield performance clearly increased when we modeled both yield F_5_ and F_6_ data simultaneously in the analysis. Furthermore, the prediction accuracy calculated from test data involving both pedigree, genomic and spatial information was clearly higher than data obtained from genomic information.

### Genetic correlation is critical for improving accuracy in MTGP

Theoretically, genetic correlation can arise mainly by pleiotropy or, less commonly, by linkage disequilibrium [34]. A high genetic correlation between two traits does not imply that both traits are highly heritable, but neither does a high phenotypic correlation [25]. Several studies using both real and simulated data have suggested that the genetic correlation between genetically-linked traits is important for multivariate genomic selection to be advantageous [13,15,20,23,25,35]. Therefore, genetic correlations between traits of interest have been recently exploited to increase the statistical power for detecting segregating QTLs [36,37] and to improve accuracy in genomic predictions in plant breeding programs [20,23,35].

To date, there have been only a few published multiple trait studies using field data for plant breeding [15–17,20,23,38]. In a simulation study, Jia and Jannink [23] reported that for two traits with no genetic correlation, the prediction accuracy of STGP was equivalent to or even better than the accuracy of MTGP. In the current study, the genetic correlation between yield in F_5_ and in F_6_ data was approximately 0.7 in both spring barley and winter wheat breeding lines. Because we collected phenotypes from different years and locations, the environmental effects on yield in F_5_ and F_6_ were independent. Our results showed that MTGP outperformed STGP by 7% of yield in spring barley and 57% in winter wheat. A similar improvement rate (60%) using MTGP was also reported from pine breeding data [23]. Notably, the predictive ability for spring barley was generally higher than it was for winter wheat, but the relative improvement was not as dramatic as it was for winter wheat. For winter wheat, the predictive ability for yield was 0.23 for F_6_ generation in our single trait analysis, and 0.37 using yield data from F_5_ and F_6_ generations in the multiple trait analysis. Because the predictive ability for spring barley was 0.48 using yield data from F6 with the STGP model, but 0.51 using MTGP, the result clearly shows that, for estimating yield performance, the prediction accuracy of the STGP model for the spring barley line was better than was the MTGP model for the winter wheat breeding lines.

### Yield heritability difference between F_5_ and F_6_

For spring barley and winter wheat, our results showed that the heritability of yield in F_5_ and F_6_ differed slightly. One reason to cause the differences could be due to the smaller plot size and lower sowing density for F_5_. In addition, the F_5_ data were tested on one location with one replicate only (compared with F_6_, there were multiple tested locations and plots), this may cause that the genetic effects included both general additive genetic effects plus specific additive genetic effects due to GxE effect between genotypes and the one location used. These effects cannot be separated for F_5_ data, compared with F_6_ data. As such, the above reason cloud lead to the heritability difference between yield F_5_ and F_6_.

### Genomic information boosts the prediction accuracy

To our knowledge, there are only few major QTLs segregating identified (such as, the *Mlo* locus), at least for the economically important traits we investigated in this study. A review by Bernardo [39] stated that approximately 10,000 QTLs have been identified by QTL mapping experiments on twelve major crop species. However, only a few QTLs have been exploited in marker-assisted selection in practical breeding schemes, which indicates that most economically important traits in spring barley and winter wheat are highly polygenic in nature. Thus, if sufficient genomic information is available (e.g., segregated SNPs across an entire genome), then genomic predictions can be an efficient tool for capturing genetic variances, much more efficient than relying on pedigree records in plant breeding. Previous studies that applied genomic-based BLUP (GBLUP) approaches show consistent prediction accuracies across various genetic architectures under simulated scenarios [40]. Additionally, Jia and Jannink [23] indicated that multiple trait GBLUP performed equally as well as Bayesian models (Bayes A and Bayes Cpi) when the traits were controlled by a polygenic genetic architecture. Both authors suggested that BLUP is likely an ideal option for modelling the traits we investigated. In this study, our model involved both genomic and pedigree information, the prediction accuracies were slightly higher than using genomic information only. This result suggests that our evaluation involving pedigree information was less accurate than using a genomic-based method. On the other hand, GBLUP is potentially not as robust as the Bayesian model when there are outliers involved (e.g., the disease traits in spring barley investigated in this study deviated from the normal distribution). The prediction accuracy reported in this study was sufficiently high (e.g., prediction accuracy > 0.5) for genomic breeders to make selection decisions on favored traits earlier in the breeding cycle, which would enable them to maximize genetic gains [17].

### Clear genetic grouping observed in commercial spring barley and winter wheat breeding lines

Although our principal components analysis (PCoA) indicated that the genetic relationship and degree of variation between all lines in both species we examined differed slightly, the PCoA clearly showed that there were some segregating groups among all breeding lines, thus implying that many lines had strong genomic relationships in certain genetic clusters.

### Future perspectives

Simulation studies based on STGP have suggested that when a high SNP marker density is used, a substantial improvement in prediction accuracy can be expected in genomic evaluations [41]. Our study used a full set of marker genotypes as well as the total available population in the MTGP model. However, genotyping cost is still a major concern in plant breeding, especially for commercial breeders. Therefore, although our approach has been tested using simulation scenarios [13], the effect of marker density and optimization of TP size may require further investigation based on real data. In addition, non-standard phenotypes, such as those obtained from metabolomics data, may assist practitioners in boosting correlations in MTGP. For example, some investigations have involved metabolomics data in multiple trait analyses, aiming to improve accuracy in plant breeding schemes (and in animal breeding) [17,42–45]. Although STGP usually achieves a predictive ability similar to MTGP in some cases (e.g., soybeans [38], bread wheat [17], durum wheat [18], and African cassava [15]), our study suggests that the predictive ability of certain traits can be improved using MTGP (based on winter wheat and spring barley breeding lines and the large number of lines we included in our study). As such, cereal breeders can apply MTGP, combined with GxE effect, to improve predictive ability for selecting high yielding cultivars with improved resistance and quality by exploiting genetic correlation between the traits.

## Conclusion

Our study showed that the MTGP approach is better than STGP for predicting yield traits in spring barley and winter wheat breeding lines when we included yield in F_5_ and yield in F_6_ in the evaluation. We also found that a model fitting pedigree, genomic and spatial information will have better prediction accuracy than using genomic information only. To conclude, prediction accuracy clearly increased in both species when we modelled yield data from F_5_ and F_6_ generations with MTGP, GxE, and spatial effects in the model. Thus, breeders can use the genetic relationship between traits to predict future trait performance, with considerably improved accuracy, by including genetically related traits using multivariate genomic prediction approaches.

## Supporting information

S1 Data (GENOTYPE)

S2 Data (YIELD)

S3 Data (PHENOTYPE)

S4 Data (GENOTYPE)

S5 Data (R)

S6 Data (DIR)

S7 Data (DIR)

S1 File (DOCX)

S1 Dataset (XLSX)

S2 Dataset (XLSX)

## Abbreviations

BLUP: Best linear unbiased prediction
GP: Genomic prediction
GS: Genomic selection
LD: Linkage disequilibrium
MAS: Marker-assisted selection
MT: Multiple trait
MTGP: Multiple trait genomic prediction
QTL: Quantitative trait loci
SNP: Single nucleotide polymorphism
ST: Single trait
STGP: Single trait genomic prediction
TP: Training population
VP: Validation population
