# Supplementary material for: Use of multiple traits genomic prediction, genotype by environment interactions and spatial effect to improve prediction accuracy in yield data
Source: PLoS One. 2020 May 13;15(5):e0232665. doi: 10.1371/journal.pone.0232665 (PMC7219756; doi:10.1371/journal.pone.0232665)
Supplement: S1 File — (DOCX) [file pone.0232665.s008.docx]

**Use of multiple traits genomic prediction, genotype by environment interactions and spatial effect to improve prediction accuracy in yield data**

Supplementary File

Environment Information in Three Experimental Locations

The field experiments were conducted on three locations in Denmark, including Dyngby, Skive, and Holeby. In terms of soil texture profile, according to Danish agricultural soil type classification (Madsen et al., 1992), Dyngby is JB6 type (10-15% clay, 0-30% silt, 40-90% fine sand, 55-90% coarse sand, and 0-10% humus), Skive is JB4 type (5-10% clay, 0-25% silt, 40-95% fine sand, 65-90% coarse sand, and 0-10% humus) and Holeby is JB7 type (15-25% clay, 0-35% silt, 40-85% coarse sand, and 0-10% humus). The yearly average temperature of Jutland region was approximately 0.5 celsius degree lower than Sealand region. The yearly average rainfall were similar, which were approximately 750 mm, in both Jutland and Sealand regions (<http://www.dmi.dk/vejr/arkiver/vejrarkiv/>).

Reference

HB Madsen, AH Nørr, and KA Holst. 1992. Atlas of Denmark Series I, Volume 3 The Danish Soil Classification, 56 p.
